# Comparative genome and phenotypic analysis of *Clostridium difficile *027 strains provides insight into the evolution of a hypervirulent bacterium

**DOI:** 10.1186/gb-2009-10-9-r102

**Published:** 2009-09-25

**Authors:** Richard A Stabler, Miao He, Lisa Dawson, Melissa Martin, Esmeralda Valiente, Craig Corton, Trevor D Lawley, Mohammed Sebaihia, Michael A Quail, Graham Rose, Dale N Gerding, Maryse Gibert, Michel R Popoff, Julian Parkhill, Gordon Dougan, Brendan W Wren

**Affiliations:** 1 London School of Hygiene and Tropical Medicine, Keppel Street, London, WC1E 7HT, UK; 2 Wellcome Trust Genome Campus, Hinxton, Cambridge, CB10 1SA, UK; 3 Hines VA Hospital, Hines, IL 60141, USA; 4 Institut Pasteur, rue du Dr Roux, 75724, Paris, France

## Abstract

**Background:**

The continued rise of *Clostridium difficile *infections worldwide has been accompanied by the rapid emergence of a highly virulent clone designated PCR-ribotype 027. To understand more about the evolution of this virulent clone, we made a three-way genomic and phenotypic comparison of an 'historic' non-epidemic 027 *C. difficile *(CD196), a recent epidemic and hypervirulent 027 (R20291) and a previously sequenced PCR-ribotype 012 strain (630).

**Results:**

Although the genomes are highly conserved, the 027 genomes have 234 additional genes compared to 630, which may contribute to the distinct phenotypic differences we observe between these strains relating to motility, antibiotic resistance and toxicity. The epidemic 027 strain has five unique genetic regions, absent from both the non-epidemic 027 and strain 630, which include a novel phage island, a two component regulatory system and transcriptional regulators.

**Conclusions:**

A comparison of a series of 027 isolates showed that some of these genes appeared to have been gained by 027 strains over the past two decades. This study provides genetic markers for the identification of 027 strains and offers a unique opportunity to explain the recent emergence of a hypervirulent bacterium.

## Abstract 2

A genome comparison of non-epidemic and epidemic strains of Clostridium difficile reveals gene gains that could explain how a hypervirulent strain has emerged

## Background

*Clostridium difficile*, a spore-forming anaerobic bacillus that often resides in the gut of mammals, is the causative agent of *C. difficile *infection (CDI) (reviewed in [1]). The hospital environment and patients undergoing antibiotic treatment provide a discrete ecosystem where *C. difficile *persists and selected virulent clones thrive. Consequently, *C. difficile *is the most frequent cause of nosocomial diarrhea worldwide, where patients exhibit a range of symptoms from mild diarrhea to life threatening pseudomembranous colitis (PMC) [2,3]. In most cases of CDI antibiotic therapies disrupt the protective gut microbiota, whereupon ingested or existent *C. difficile *spores germinate, colonize the gastrointestinal tract and produce toxins. Another feature of CDI is the high relapse rate due to re-infection or reactivation of infection [2,3]. The population at risk for CDI includes not only patients on antimicrobial and other therapies that can alter the balance of the gut microbiota (for example, antacid/proton pump inhibitors and non-steroidal anti-inflammatory drugs), but also the immunocompromised and the elderly. Given the continued use of broad-spectrum antibiotics and the rising numbers of immunocompromised and elderly patients, the problems associated with CDI are unlikely to recede.

Alarmingly, in the past 5 years a new group of highly virulent *C. difficile *strains has emerged to cause outbreaks of increased disease severity in North America and Europe. Several studies have shown that patients infected with these PCR-ribotype 027 strains have more severe diarrhea, higher mortality and more recurrences [4-8]. Prior to 2003, only a handful of these strains were isolated in the UK, whereas currently most typed isolates are PCR-ribotype 027. This is also mirrored in Canada, where 027 strains were undetected in 2000, but reached 75.2% of all PCR-ribotyped strains by 2003 [9]. The emergence of 027 strains might partially explain the 72% annual increase in mortality in the UK due to CDI to 6,500 cases in 2006 [7]. The CDI outbreaks at the Stoke Mandeville hospital, Buckinghamshire, marked the arrival of the epidemic 027 isolates to the UK. Between April 2003 and March 2006 a total of 498 patients acquired *C. difficile *at the hospital (measured by onset of symptoms 72 hours after admission), of which 127 died [10].

PCR-ribotype 027 strains are genetically highly uniform, which is confirmed by the application of diverse genotyping methods. For example, 027 strains are invariably designated as BI by restriction endonuclease analysis, NAP1 (North American pulsotype 1) by pulse field gel electrophoresis, are exclusively toxinotype III by toxinotyping and are indistinguishable by multi-locus sequence analysis [11]. The earliest retrospective recorded PCR-ribotype 027 isolate was strain CD196 in 1985, which is a non-epidemic strain isolated from a single patient with CDI in a Paris hospital [12]. The next retrospective recorded 027 isolate was a non-epidemic strain designated BI-1, which was from a patient with CDI in a Minneapolis hospital in 1988 [13]. Since 1988 a further 19 BI designated strains (all PCR-ribotype 027) have been isolated and characterized by Gerding and colleagues representing a useful time-line of the evolution of 027 strains (DN Gerding, personal communication).

Comparative phylogenomics (whole genome comparisons of bacteria using DNA microarrays combined with Bayesian-based algorithms to model the phylogeny) was recently applied to 75 *C. difficile *strains of diverse origin, including 19 strains confirmed as PCR-ribotype 027 (16 BI strains from the US, CD196, a strain from a recent Canadian outbreak and a representative strain from the Stoke Mandeville outbreak designated R20291). All 027 strains formed a tight clade, which was distinct from the other 56 strains analyzed [14]. Closer inspection of the 027 clade revealed micro-evolution among strains with the historic non-epidemic CD196 and BI-1 strains as progenitors compared to their recently isolated counterparts [14]. These studies confirm the clonal nature of PCR-ribotype 027 strains and that they are continuing to evolve.

*C. difficile *is known to produce two related glucosylating toxins, named toxin A and toxin B, which are encoded on the pathogenicity locus (PaLoc) [15]. For some time, toxin production has been the main focus of study when addressing virulence of *C. difficile*. However, in the hamster model of infection toxin B plays the most significant role in infection [16]. A recent report has shown the binding domain of toxin B in 027 strains to be highly divergent compared to other *C. difficile *strains [8]. However, the significance of the difference of the 027 toxin B gene sequence has yet to be investigated. The PaLoc also includes toxin regulatory components, including *tcdR*, a sigma factor, and *tcdC*, a negative regulator that destabilizes the TcdR-holoenzyme to prevent transcription of the PaLoc [17]. It has been reported that some 027 strains can produce more toxin *in vitro *[18], which was initially attributed to deletions in the negative regulator *tcdC*. Further characterization has revealed that the 18-bp in-frame deletion was found to have no effect on toxin production [19]. Two additional deletions have been identified within *tcdC*, a 39 and single base-pair deletion. The single base-pair deletion results in the formation of a stop codon downstream and truncation of the protein, thus leading to increased toxin production. However, various deletions have been identified in *tcdC *in non-epidemic PCR-ribotypes as well [20], suggesting the increased virulence cannot solely be attributed to these deletions. This has stimulated debate on the mode of hypervirulence in the epidemic 027 strains. Apart from classic virulence determinants such as toxin production, other factors such as antibiotic resistance, increased motility and adherence in the gut, increased resistance to bile salts and increased transmissibility manifested through sporulation might explain the emergence of epidemic 027 strains. A recent report comparing three 'historical' 027 strains from Sweden with an epidemic strain concluded that the epidemic strain sporulated more readily than its three non-epidemic counterparts [21].

Given the medical and economic importance of CDI and the difficulties in studying the genetics of *C. difficile*, we recently reported the complete genome sequence of a pathogenic *C. difficile *strain [22]. The strain chosen, 630 (PCR-ribotype 012), was a multi-drug-resistant isolate from a patient with PMC at a hospital in Zurich in 1982 [22]. The full sequence revealed a 4.29 Mb chromosome with a mosaic of potential mobile genetic elements, antibiotic resistance genes and virulence determinants [22].

The rapid international emergence of the *C. difficile *027 strain lineage provides a unique opportunity to understand the recent emergence of a highly virulent bacterium. In this study we undertake a three-way genome comparison of an 'historic' non-epidemic 027 *C. difficile *strain (CD196), a recent epidemic and hypervirulent 027 strain (R20291) and the previously published PCR-ribotype 012 strain (630). Where possible we relate genetic differences to phenotypic differences observed in these strains with respect to motility, survival, antibiotic resistance and toxicity.

## Results and discussion

### Genome comparison of the PCR-ribotype 027 strains (CD196 and R20291) and strain 630

**Figure 1:**
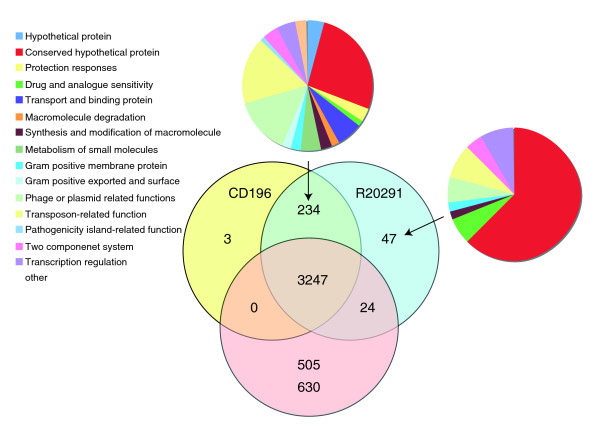
**Distribution of orthologues CDSs in *C. difficile *strains 630, CD196 and R20291**. The Venn diagram shows the number of genes unique, shared or core between the three strains. The associated pie charts show the breakdown of the functional categories assigned to these CDS.

**Figure 2:**
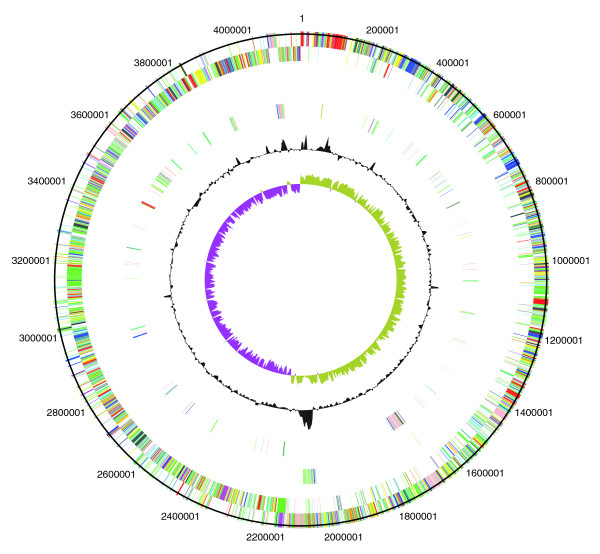
**Circular representations of *C. difficile *chromosomes**. From the outside (scale in bp): circles 1 and 2 show the position of R20291 CDS transcribed in a clockwise and anti-clockwise direction colored according to predicted function; circle 3 shows CDS unique to R20291; circle 4 shows CDS unique to both R20291 and CD196; circle 5 shows GC content; circle 6 shows GC deviation (> 0%, olive; < 0%, purple). Color coding for CDS functions: dark blue, pathogenicity/adaptation; black, energy metabolism; red, information transfer; dark green, surface-associated; cyan, degradation of large molecules; magenta, degradation of small molecules; yellow, central/intermediary metabolism; pale green, unknown; pale blue, regulators; orange, conserved hypothetical; brown, pseudogenes; pink, phage and IS (Insertion Sequence) elements; grey, miscellaneous.

The two newly sequenced genomes of the PCR-ribotype 027 strains (CD196 historic and R20291 modern; Table 1) were compared with the previously sequenced strain 630 (PCR-ribotype 012). The three strains share 3,247 core genes, including those encoding determinants important for pathogenesis, such as antimicrobial resistance, ethanolamine/propanediol metabolism, sporulation, a beta-lactamase-inducing penicillin-binding protein, a quaternary ammonium compound-resistance protein, tellurium resistance proteins, a putative nogalamycin resistance protein and L-rhamnose biosynthesis (Figure 1). There are 505 coding sequences (CDSs) unique to 630 compared to the 027 strains, whereas there are 47 CDSs unique to R20291 and three CDSs unique to CD196 (Figure 1). The locations of regions of genetic difference between the three strains are highlighted in the concentric circular chromosome representations of the three genomes (Figure 2). There are 234 genes unique to both 027 ribotypes spread among at least 50 regions of genetic difference (Figure 2; Additional data file 1). These include a phage island, transposon genes, two-component response regulators, drug resistance genes, transporter genes and type I restriction enzyme/restriction modification genes (Additional data file 1).

**Table 1:** Strains used in this study

**Isolates**	**Date isolated/recorded**	**City, state/province**
630	1982	Zurich, Switzerland
CD196	1985	Paris, France
R20291	2006	Aylesbury, UK
BI-1	26/2/1988	Minneapolis, MN
BI-2	14/1/1991	Tucson, AZ
BI-3	14/12/1990	Minneapolis, MN
BI-4	10/2/1993	Minneapolis, MN
BI-5	25/8/1995	Albany, NY
BI-6	20/5/2003	Portland, OR
BI-6p	09/9/2004	Atlanta, GA
BI-6p2	09/9/2004	New Jersey
BI-7	20/5/2003	Portland, OR
BI-8	22/1/2004	Portland, ME
BI-10	10/8/2001	Pittsburgh, PA
BI-11	10/8/2001	Pittsburgh, PA
BI-12	09/9/2004	Camp Hill, PA
BI-13	09/9/2004	New Jersey
BI-14	09/9/2004	New Jersey
BI-15	09/9/2004	New Jersey
BI-16	01/9/2004	Augusta, ME
BI-17	05/10/2004	Montreal, Quebec

There are 14 CDSs that have been disrupted by an insertion in both 027 strains but are intact in 630; conversely, 12 CDSs are intact in both 027 strains but have been disrupted in 630 (Additional data file 2). All three genomes have multiple copies of genes for transposase-like proteins that have been inserted both intragenically and intergenically. In *C. difficile *630 there are eight full transposon copies and two remnant copies; all eight functional copies have inserted within CDSs. In both 027 strains there are 17 transposon copies, of which only 6 inserted within CDSs. Only three CDSs are interrupted by transposons in all three strains. Furthermore, three CDSs have been truncated by sequence loss in both 027 strains but are intact in 630 and 10 CDSs are truncated in 630 but not 027 strains (Additional data file 2). Finally, point mutations have resulted in frameshifts exclusively in three 630 CDSs and 10 of the 027 strain CDSs (Additional data file 2).

### Toxin-related genes specific to 027

Variation within the PaLoc region (containing toxins A and B and their associates genes) [15] between *C. difficile *strains has been observed frequently and has been used to develop the toxinotyping method to distinguish strains [23-25]. PCR-ribotype 027 isolates are invariably toxinotype III, whereas 630 (PCR-ribotype 012) is toxinotype 0. A comparison of the PaLoc sequences from 630, R20291 and CD196 confirms the previous data, indicating that the *tcdB *sequence varies among strains, particularly at the 3' region, which encodes the toxin-binding domain [26]. However, there is a high level of sequence conservation in *tcdB *between the 027 strains CD196 and R20291 compared to strain 630. Examination of the relative *in vitro *cytotoxicity of these three strains on several cell lines confirms differences in both toxicity and cell line specificity (Table 2). Purified toxin B from R20291 has more potent activity than that from 630 in all eight cell lines tested whereas the historic 027 (CD196) is more potent in six of the eight cell lines tested (Table 2). Given the recent demonstration in the hamster model of CDI that toxin B, and not toxin A, is essential for virulence, the observation that toxin B from strain R20291 has a more potent activity over a broader range of cell types may indicate that this is a contributory factor to the hypervirulence of R20291 [16].

**Table 2:** Toxin B cytotoxicity assay

	**630**	**CD 196**	**R 20291**
VERO	2.00E-07	1.60E-09	1.00E-09
HELA	3.00E-06	1.00E-08	4.00E-10
3T3	3.00E-06	1.00E-08	4.00E-10
NG108	2.50E-04	3.80E-06	6.00E-08
MDCK	9.00E-05	3.00E-05	1.60E-06
CaCO2	9.00E-05	3.00E-05	1.20E-07
Hep2	1.00E-06	3.50E-08	4.00E-10
CHO	1.20E-05	1.24E-08	1.00E-08

Cytotoxicity as expressed as toxin molarity (M) that induces 50% cell rounding after 24 hour exposure.

Regulation of toxin expression has also been shown to vary between strains of *C. difficile*, which has been attributed to mutations in the negative regulator *tcdC *[9]. The most notable of these mutations is the 1-bp deletion present in 027 strains that results in a frame shift and truncation of TcdC, causing de-repression of the PaLoc [17]. A single base deletion at position 117, resulting in truncation of TcdC at the 66th amino acid, was confirmed in both R20291 and CD196 but absent from 630. The presence of the 18-bp deletions in both R20291 and CD196 but their absence from 630 was confirmed.

The binary ADP-ribosyltransferase toxin, first identified in 1988 in the strain CD196 [12], consists of two genes, *cdtA *and *cdtB*. Surveys have identified the binary toxin in up to 8.6% of *C. difficile *strains [27-30]; recently, however, binary toxin positive PCR-ribotype 027 incidence has reached 41.3% in the UK [31]. Additionally, the binary toxin has been linked with increased severity of disease [32-34]. Sequence analysis confirms the presence of full-length *cdtA *and *cdtB *genes in both CD196 and R20291, which by contrast have accumulated sequence deletions, several frameshift mutations and in-frame stop codons in 630. Recently, the CDS upstream has been identified as the binary toxin response regulator, designated *cdtR *[35]. *C. difficile *630 contains a functional copy of *cdtR *despite lacking binary toxin, and CdtR is 96% identical to the homologues found in both 027 isolates.

### Differences in antibiotic resistance between 630 and PCR-ribotype 027 strains

In contrast to strain 630, the epidemic 027 strains are highly resistant to fluoroquinolones due to point mutations in the DNA gyrase genes [36]. Comparison of the *gyrA *gene identified seven point mutations in DNA gyrase genes between *C. difficile *630 and both 027 strains. Four mutations are silent and two substitutions - Leu406Ile and Asp468Asn - conserved. Interestingly, the previously described Thr82Ile conversion was only present in the epidemic 027 [36]. Two silent point mutations (A1458G and C1890T) were identified in the *gyrB *gene of the 027 strains. Three fluoroquinolones (gatifloxacin, moxifloxacin and lexofloxacin) were used and results showed that R20291 was highly resistant to the fluoroquinolones (≤ 32 mg/l for all three fluoroquinolones), but CD196 was fluoroquinolone sensitive (gatifloxacin minimum inhibitory concentration (MIC) 1.5 mg/l, moxifloxacin MIC 2 mg/l and lexofloxacin MIC 3 mg/l) and 630 was sensitive or had intermediate resistance to fluoroquinolones (gatifloxacin MIC 2 mg/l, moxifloxacin MIC 1.5 mg/l and lexofloxacin 6 mg/l).

Sequence data revealed that both 027s have acquired two unique conjugative transposons absent in *C. difficile *630. One of these transposons (CTn-*027*) encodes a novel chloramphenicol resistance gene (CDR20291_3461). R20291 and CD196 demonstrated intermediate resistance (MIC 16 mg/l), but 630 was sensitive to chloramphenicol (MIC 12 mg/l) (*P *< 0.05).

*C. difficile *strain 630 has two copies of the erythromycin resistance gene (*ermB1*/CD2007 and *ermB2*/CD2010) on a mobile transposon Tn*5398 *(CD2001-2010b), which was absent in CD196 and R20291. However, experimental data showed that *C. difficile *630 and R20291 were erythromycin resistant (MIC ≥ 256 mg/l) whereas CD196 had intermediate resistance (MIC 2 mg/l). Additionally, strain 630 has a tetracycline resistance gene (*tetM*; CD0508) on CTn*3*/Tn*5397 *(CD0496-511), which is absent in CD196 and R20291. *C. difficile *630 demonstrated tetracycline resistance (MIC 64 mg/l) in contrast to CD196 and R20291, which were tetracycline sensitive (MIC 0.17 mg/l and 0.22 mg/l, respectively) (*P *< 0.001).

The difference between drug resistance patterns may reflect changes in antibiotic policy. For example, both CD196 and 630 predate 1992 when Golledge *et al*. [37] demonstrated clindamycin not to be a risk factor; subsequently, clindamycin use has been strongly associated with PCR-ribotype 027 outbreaks [38-40]. This demonstrates that antibiotic usage may be driving the evolution of drug resistance and the predominance of certain isolates.

### 027-specific genes involved in flagella biosynthesis, glycosylation and motility

Flagella have been found to be important for motility in several enteric pathogens as a prerequisite to traverse the mucous layer of the gut to interact with gut epithelial cells [41-43]. Additionally, chemotaxis mediated through motility is important in survival, to enable movement towards nutrient-rich sources and movement away from noxious environments. Flagella have been observed in some *C. difficile *strains [44,45]. Post-translational modification of flagellin proteins by glycosylation has been shown to be prevalent in several bacterial pathogens and the loci encoding these modifications are frequently located adjacent to the structural flagellin genes [46]. Such modifications are important in subverting host immune defenses [47], autoagglutination [48] and adhesion and colonization [49].

**Figure 3:**
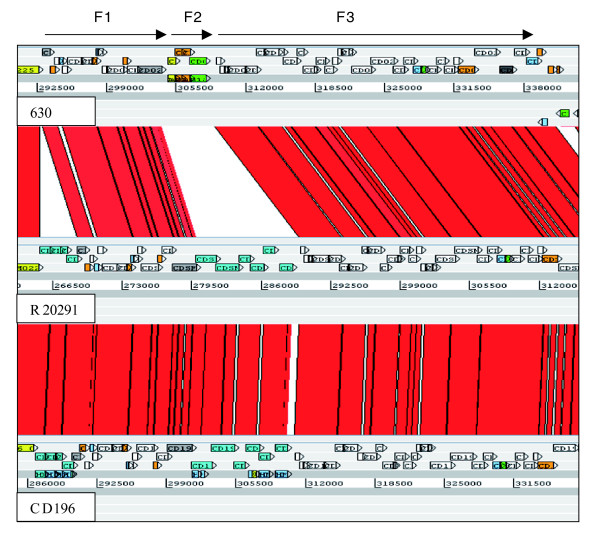
**ACT comparison of flagellin and flagellin glycosylation-associated loci**. F1 genes are CD0226-240 (630), CDR20291_0227- 241 (R20291), and CD196_0240-254 (CD196). F2 genes are CD0241-244 (630), CDR20291_0242-247 (R20291), and CD196_0255-260 (CD196). F3 genes are CD0245-271 (630), CDR20291_0248-275 (R20291), and CD196_0261-288 (CD196). Red bars indicate > 84% DNA sequence identity.

In 630, flagella-associated genes are found in two loci, F1 (CD0226-CD0240) and F3 (CD0245-CD0271), which are separated by an inter-flagella locus F2 (CD0241-CD0244). Loci F2 encodes a phosphoserine phosphatase, two conserved hypothetical proteins and a putative CDP-Glycerol:Poly (glycerophosphate) glycerophosphotransferase [14] (Figure 3). Microarray analysis of this region previously showed a loss of, or high divergence in, F1 and F2 in all 027 isolates tested [14]. The sequence data from both R20291 and CD196 show that the F1 locus has been retained, but with only 84 to 90% sequence identity, whereas the four genes present in the inter-flagella F2 locus of 630 have been replaced by six different genes encoding a glycosyl transferase (family 2), two putative uncharacterized proteins, a putative carbamoyl-phosphate-synthetase and a putative ornithine cyclodeaminase (Figure 3).

**Figure 4:**
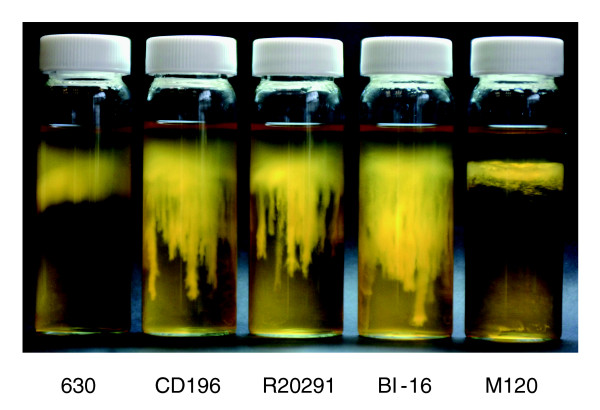
**Comparative motility assays for *C. difficile *strains**. The motility of strain 630 was compared to that of both recent and historic 027 ribotypes, R20291, BI-16 and CD196; M120 was the non-motile control. Strains were inoculated into 0.05% BHI agar and incubated for 24 hours in an anaerobe chamber. The motility is visualized as stalactite projections.

The variation in the F1 region between 630 and the 027 ribotypes may be important in motility, as there are clear phenotypic differences in the motility of 630 and the 027 ribotypes CD196, R20291 and BI-16 (Figure 4). *C. difficile *630 is less motile than the 027 ribotypes, whereas M120 is non-motile (Figure 4). Microarray data have shown the absence/divergence of the complete F3 region in M120 [14]. Recent sequence data for M120 have confirmed the deletion of the entire F3 region in this strain [50], explaining the lack of motility for strain M120. The subtle differences in motility between the 630 and the 027 ribotypes may be due to the levels of sequence conservation over the F1 region.

**Figure 5:**
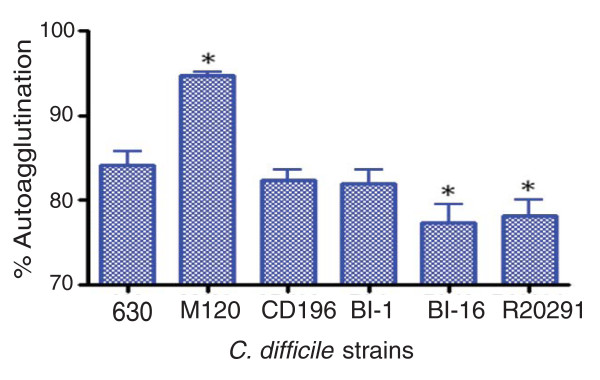
**Autoagglutination of *C. difficile *strains**. *C. difficile *strains were grown on BHI plates for 1 to 2 days, then inoculated into pre-equilibrated phosphate-buffered saline to an OD600 nm of 1.0 (± 0.1). These were incubated for 24 hours in pre-equilibrated glass tubes, then the OD600 nm was measured. The percentage of autoagglutination was normalized to the starting OD ((Starting OD - Final OD)/Final OD × 100). The bars indicate the percentage of cells autoagglutinating. Significant differences in autoagglutination are marked with an asterisk; *P *< 0.05, Students *t*-test. M120 is a non-motile strain thar autoagglutinates to a significantly higher level than 630 (*P *< 0.05).

The different genes present in the F2 region of 630 and the 027 ribotypes may be important in the glycosylation of the flagella, as the six genes present in R20291 and CD196 contain glycosyl transferases. Studies in other enteric bacteria such as *Campylobacter jejuni *have shown that both Flagellin, encoded by *FlaA*, as well as post-translational modifications of it are required for autoagglutination, which is linked to virulence [48]. Significant differences in autoagglutination between 630 and the more recent 027 isolates R20291 and BI-16 (*P *< 0.05) were observed, whereas the more historic 027 isolates BI-1 and CD196 show no significant difference in autoagglutination compared to 630 (Figure 5). The differences in autoagglutination observed between 630 and the recent 027 isolates are likely to be multifactorial as, in addition to flagella and glycosylation, other surface phenomena can contribute to autoagglutination.

Four 027 unique genes upstream of the flagella F1 region (CDR20291_0223-0226 and CD196_0236-0239) that are absent in 630 may be involved in virulence. The four CDSs encode DTDP-4-dehydrorhamnose reductase, glucose-1-phosphate thymidylyltransferase, DTDP-4-dehydrorhamnose 3,5-epimerase and DTDP-glucose 4,6-dehydratase. These four enzymes (RlmA, B, C and D) are involved in the synthesis of L-rhamnose. Carbohydrates such as L-rhamnose can act as structural elements as well as energy sources [51] and can be important virulence factors in both Gram-positive and Gram-negative bacteria. In *Vibrio cholerae*, *Escheichia coli *and *Salmonella enterica*, L-rhamnose is an important residue in the O-antigen of lipopolysaccharides. In *Streptococcus *mutans, L-rhamnose is part of an antigen involved in colonization of tooth surfaces [52] and mutations in this pathway have been shown to prevent initiation and maintenance of infection [53]. In *Mycobacterium tuberculosis*, L-rhamnose links peptidoglycan and arabinogalactan to form the unique cell wall. Given their co-location in the F regions, it is possible that these genes may play a role in flagellin glycosylation in the 027 strains.

### 027 specific regulatory genes that may be important in survival

Regulatory genes form a large proportion of the 027-specific genes, with 8 two-component regulators and 15 other transcriptional regulators. One of the most striking regions of genetic difference was an additional complete copy of the *agr *regulatory locus (termed *agr2*), consisting of *agrA *(CDR20291_3189/CD196_3143), *agrB *(CDR20291_3187/CD196_3141), *agrC *(CDR20291_3188/CD196_3142) and *agrD *(CDR20291_3187a/CD196_3141a). The *agr1 *locus from *C. difficile *630 contains only single copies of *agrB *and *agrD*, with the response regulator (*agrA*) and histidine protein kinase (*agrC*) genes absent. The *agr1 *locus was present in both 027 strains. The complete *agr *locus (*agrA *to *agrD*) has been identified as a key regulatory system involved in multiple aspects of virulence and quorum sensing in *Staphylococcus aureus *[54]. Downstream of the *agr2 *locus are three 027-specific CDSs that encode two putative membrane proteins and an ABC transporter ATP-binding protein.

One of the additional transcriptional regulators in the 027 ribotypes is a PadR-like transcriptional regulator (CDR20291_2964/CD196_2917). The PadR family regulates phenolic acid metabolism, which may be important in survival of bacteria in the gut, where energy sources are limited. The CDS is found within a region of six 027-specific genes - transcribed on the opposite strand to the other five CDSs - that encode a predicted enoate reductase, a nitrate/nitrite transporter and a conjugative transposon site-specific recombinase. The PadR regulator may also be important in tolerance or production of *p*-cresol, a phenolyic agent produced by *C. difficile *from the degradation of tyrosine. The *p*-cresol operon CD0153-155 was conserved within both 027s and in 630. However, there are clear phenotypic differences between the tolerance to *p*-cresol between the recent 027 isolates and 630 [55], which may be due to PadR or another transcriptional regulator.

### Genetic differences between the historic CD196 strain and the R20291 hypervirulent strain

**Figure 6:**
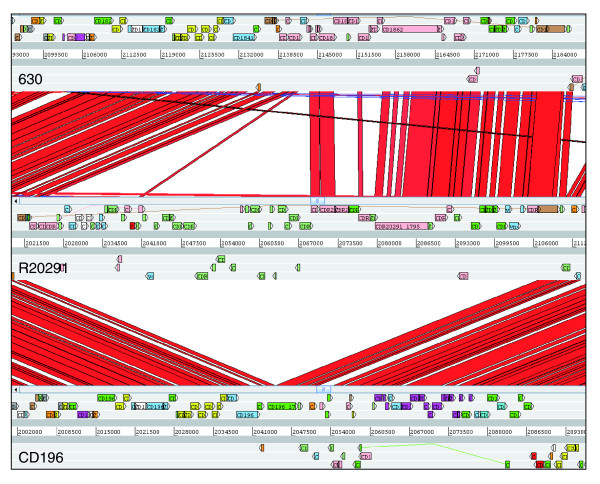
**Comparison of phage island SMPI (Stoke Mandeville phage island) between *C. difficile *strains 630, CD196 and R20291**.

Sequence data show that there are at least five genetic regions unique to the epidemic 027 (R20291) compared to the non-epidemic 027 strain (CD196) (Table 3). We hypothesize that these newly identified R20291 genetic elements contribute to the virulent phenotype of this clone. These genetic regions include a unique approximately 20-kb phage island of high G+C DNA content termed SMPI1 inserted into a 027 unique conjugative transposon (named CTn*027*; Figure 6). This phage island insertion sequence disrupts the R20291 CDS CDR20291_1744 and carries a number of cargo genes present only in R20291, including a two-component response regulator (CDR20291_1748), a putative lantibiotic ABC transporter (CDR20291_1752), a putative cell surface protein along with a number of hypothetical and conserved hypothetical proteins. CDR20291_1755 is a unique R20291 gene encoding a transcriptional regulator (σ^24^). The phage island also encodes a toxin-antitoxin system (RelE/StbE family) that is important in maintaining the stability of mobile elements [56]. RelE encodes a stable toxin that inhibits translation by cleaving mRNAs on translating ribosomes [57]. The toxin is inhibited by an unstable anti-toxin (RelB). This toxin-antitoxin system has been linked to translation moderation under amino-acid starvation stress [58].

**Table 3:** 

**R20291**	**Function**
CDR20291_0183	Putative membrane protein
CDR20291_1419	Uncharacterized protein
CDR20291_1744	Site-specific recombinase
CDR20291_1745	Uncharacterized protein
CDR20291_1746	Uncharacterized protein
CDR20291_1747	Putative conjugative transposon regulatory protein
CDR20291_1748	Two-component response regulator
CDR20291_1749	Sensor protein
CDR20291_1750	Putative lantibiotic ABC transporter, ATP-binding protein
CDR20291_1751	Putative lantibiotic ABC transporter, permease protein
CDR20291_1752	Putative lantibiotic ABC transporter, permease protein
CDR20291_1753	Uncharacterized protein
CDR20291_1754	RNA polymerase, sigma-24 subunit, ECF subfamily
CDR20291_1755	Sigma-24 (feci)
CDR20291_1756	RNA polymerase, sigma-24 subunit, ECF subfamily
CDR20291_1757	Uncharacterized protein
CDR20291_1758	Uncharacterized protein
CDR20291_1759	Addiction module toxin, RelE/StbE family
CDR20291_1760	Addiction module antitoxin, RelB/DinJ family
CDR20291_1761	Uncharacterized protein
CDR20291_1762	Phage protein
CDR20291_1763	Replicative DNA helicase
CDR20291_1764	Uncharacterized protein
CDR20291_1765	Uncharacterized protein
CDR20291_1766	Transcription regulator (yobD protein)
CDR20291_1767	Uncharacterized protein
CDR20291_1768	Uncharacterized protein
CDR20291_1769	Uncharacterized protein
CDR20291_1770	Uncharacterized protein
CDR20291_1771	Uncharacterized protein
CDR20291_1772	Uncharacterized protein
CDR20291_1773	Uncharacterized protein
CDR20291_1774	Uncharacterized protein
CDR20291_1775	Uncharacterized protein
CDR20291_1776	Putative conjugal transfer protein (putative single-stranded DNA binding protein)
CDR20291_1777	Uncharacterized protein
CDR20291_1778	Uncharacterized protein
CDR20291_1779	Uncharacterized protein
CDR20291_1780	Uncharacterized protein
CDR20291_1781	Uncharacterized protein
CDR20291_1782	Uncharacterized protein
CDR20291_1783	Uncharacterized protein
CDR20291_1784	Uncharacterized protein
CDR20291_1785	Uncharacterized protein
CDR20291_1786	Uncharacterized protein
CDR20291_1787	Uncharacterized protein
CDR20291_1788	Uncharacterized protein
CDR20291_1809	Site-specific recombinase

Both 027 strains share a similar prophage (prophage phi-027), which has integrated between the orthologues of 630 CDSs CD1566-7. These prophages (CDR20291_1415-1465, CD196_1438-89) are identical apart from one small region. CD196 contains three strain-specific adjacent CDSs, the only CD196-specific CDS in the whole genome, which encode a putative phage anti-repressor and two putative uncharacterized proteins. R20291 appears to have lost these three CDSs and replaced them with a single putative uncharacterized protein that has 88% identity at the 5' end to one of the lost uncharacterized proteins and may represent a pseudogene. In addition, there is a unique R20291 region encoding six genes, including *matE *(CDR20291_1779), a member of the Multi-antimicrobial extrusion family drug/sodium antiporters. This region also shows a high G+C content, indicating recent acquisition.

### Acquisition of R20291-specific genes in other PCR-ribotype 027 strains over time

In order to validate the presence of the R20291-specific genes and to monitor their acquisition over time, PCR analysis was undertaken on 19 PCR-ribotype 027 strains that have been isolated over a 16-year period across the US (Table 1). These isolates were typed by restriction endonuclease analysis as BI, which is equivalent to PCR-ribotype 027; however, each isolate represents a unique small variation found in the BI restriction endonuclease analysis patterns. Strains BI-1 to -5 are considered 'historic' and were isolated between 1988 and 1995. BI-6 to -17 are considered 'modern' and were isolated from 2001 to 2004. Strains 630 and CD196 (ribotypes 012 and 027, respectively) were used as negative controls (Table 4).

**Table 4:** Presence of R20291 specific genes in a time course of 19 PCR ribotype 027 strains designated BI-1 to -17

**SM unique**	**630**	**SM**	**CD196**	**1**	**2**	**3**	**4**	**5**	**6**	**6p**	**6p2**	**7**	**8**	**10**	**11**	**12**	**13**	**14**	**15**	**16**	**17**
1419		X		X	X			X	X	X	X	X	X	X	X	X	X	X	X	X	X
1744		X																			
1751*		X												X						X	
1752*		X												X	X	X	X		X	X	
1753*		X												X							
1766		X								X			X			X				X	X
1784		X																			
1788		X																			
1772		X								X						X				X	
1775		X								X			X			X	X	X		X	X
1779		X																			

*Phage island. X = PCR positive, blank = PCR negative. 630 = *C. difficile *630 sequence strain, SM = R20291 (epidemic 027), CD196 = original, non-epidemic 027, BI-1 to -5 = 'historic' ribotype 027 strains, BI-6 to -17 = 'modern' ribotype 027 strains.

Eleven R20291-specific genes were chosen for PCR analysis (Table 4). Four genes (CDR20291_1744, CDR20291_1751 to _1753) are found on the R20291-specific phage island; gene CDR20291_1744 is a site specific recombinase, CDR20291_1751 and CDR20291_1752 are putative lantibiotic ABC transporters and CDR20291_1753 is unknown. In addition, the R20291-specific transcriptional regulator (YobD protein) is also present in the 'modern' BI strains (6p, 8, 12, 16 and 17) but absent from the earlier BI strains. Only one R20291-specific gene (CDR20291_1419; BRO protein family) was amplified in the early BI strains (BI-1, -2 and -5), showing the acquisition of R20291 genes was more prevalent in the epidemic 027 BI strains (Table 4). Furthermore, recent data demonstrate that the epidemic 027 strain, named BI-6, is more virulent in the hamster infection model than early strains such as BI-1 [13].

## Conclusions

*C. difficile *is the most frequent cause of nosocomial diarrhea worldwide, in part due to the rapid and dramatic worldwide emergence of the PCR-ribotype 027 strains. We show that 027 strains have considerable genetic differences compared to strain 630 that may relate to observed phenotypic differences in motility, survival, antibiotic resistance and toxicity. Additionally, five genetic regions appear to have accumulated in the modern day epidemic 027 strain R20291 compared to the historic CD196 counterpart. This includes a unique approximately 20-kb phage island of high G+C content DNA (SMPI1) inserted into a 027 unique conjugative transposon. However, the role of individual determinants through mutagenesis and the testing of mutants in appropriate *in vivo *models is required to provide conclusive evidence. Some of these elements appear to have accumulated in 027 strains over the past 16 years and may therefore be useful genetic markers for epidemic 027 strains. The observed gene differences between these strains might individually or collectively explain why modern 027 strains are more likely to be epidemic and could explain the higher case-fatality ratio and persistence associated with infection by these strains. These studies facilitate pinpointing the genetic and phenotypic attributes that may explain the emergence of the hypervirulent 027 strain and contribute in general to our understanding of the evolution of bacterial virulence.

## Materials and methods

### Bacterial strains and growth conditions

*C. difficile *027 isolates designated BI-1 to -17 were provided by Dale Gerding (Hines VA Hospital, Hines, IL, USA). *C. difficile *630 [59] was isolated from a patient with PMC in Zurich, 1982 and has been fully sequenced by the Wellcome Trust Sanger Institute [22]. Strain 630 was provided by Peter Mullany, Eastman Dental Institute, London, UK. The 027 strain CD196 is a non-epidemic strain isolated from a patient with PMC in Paris, 1985 and was provided by Michel Popoff, Institut Pasteur, Paris, France. The hypervirulent 027 R20291 was isolated from a recent outbreak in Stoke Mandeville, UK and was provided by Jon Brazier, Anaerobe Reference Laboratory, Cardiff, UK.

*C. difficile *was routinely cultured on Braziers agar (Bioconnections, Leeds, South Yorkshire, UK) containing 4% egg yolk, *C. difficile *supplement (Bioconnections) and 2% defibrinated horse blood or in brain heart infusion (BHI) broth containing *C. difficile *supplement (Oxoid, Basingstoke, Hampshire, UK) and 0.04% cysteine. All cultures were grown in an anaerobic atmosphere (10% CO_2_, 10% H_2_, 80% N_2_) at 37°C.

### DNA isolation and PCR amplification

Genomic *C. difficile *DNA was isolated by cell lysis, phenol chloroform extraction and ethanol precipitation. Briefly, overnight cultures were resuspended in 3 ml EDTA and incubated at 37°C for 1 hour with 20 mg/ml lysozyme (Sigma-Aldrich, Gillingham, Dorset, UK), 10 KU/ml mutanolysin (Sigma-Aldrich), 5 mg/ml lysostaphin (Sigma-Aldrich) and 100 mg/ml RNase (Invitrogen, Paisley, Renfrewshire, UK). Proteinase K (25 mg/ml; Sigma-Aldrich) and 20% SDS (Sigma-Aldrich) were added to the cell suspension and incubated at 50°C for 1 hour. DNA was extracted by phenol:chloroform:IAA (Sigma-Aldrich) washes and chloroform:IAA (Sigma-Aldrich) washes. Genomic DNA was precipitated using 100% ethanol and purified with two washes of 80% ethanol. Purity was assessed and quantification done using a NanoDrop1000 spectrophotometer and by running the samples on 1.0% agarose gel, 100 mV for 45 minutes.

PCR amplifications were performed using primers described in Additional data file 3. Reactions were performed using 35 cycles at 94°C for 15 seconds, 50°C for 1 minute, 72°C for 1 minute, followed by a final extension of 72°C for 7 minutes. PCR products were analyzed on 1% agarose gels run at 100 mV for 1 hour and stained with ethidium bromide.

### DNA sequencing and assembly

Genomic sequences were generated by combining data from 454/Roche technology (using GS20 for R20291 and FLX for CD196) with shotgun capillary reads from ABI 3730xl analyzers (Table 5). Reads from the 454 platform were assembled *de novo *(without guidance from a reference sequence) into contigs using newbler (Roche, Welwyn Garden City, Hertfordshire, UK), then shredded into artificial reads of comparable lengths to capillary reads. An assembly was created with data from both platforms using Phrap. For each combined assembly the order of contigs was estimated by comparing them to strain 630 genomic sequence using ABACAS [60]. To further correct homopolymer tract errors inherent in early 454 sequencing data, Solexa (Illumina, Saffron Walden, Essex) sequence data were generated for isolate R20291. The Illumina sequences were assembled *de novo *using Velvet [61] and the resulting contigs were incorporated with the combined 454 and capillary assembly. Closing gaps between contigs for both CD196 and R20291 was either by primer walking on subclones from the capillary shotgun or by sequencing PCR products covering gaps between adjacent contigs. The final contiguous sequence for CD196 was mostly from combined data but small regions were covered with only 454 data (a total of less than 2.6% of the sequence) or with only capillary reads, giving a consensus confidence of < 41 (< 0.3% of the sequence), and rRNA repeats were represented as consensus sequences (Table 5). All regions of the final finished R20291 assembly are covered by high quality capillary reads or by combinations of data from at least two sequencing technologies, although three gaps remain where ribosomal rRNA operons have not been bridged by read-pairs. All regions of the final finished R20291 assembly are covered by high quality capillary reads or by combinations of data from at least two sequencing technologies.

**Table 5:** Average sequence coverage from aligned reads

**Isolate**	**Average 454 coverage**	**Average capillary coverage**	**Average Illumina coverage**
CD196	13.6×	5.1×	N/A
R20291	14.8×	5.7×	132.5×

### Genome annotation, comparison and orthologue identification

Genome annotation of *C. difficile *strains CD196 and R20291 was based on previously published annotations of *C. difficile *strain 630 [22]. The genomic sequences of strains CD196 and R20291 were compared against the database of strain 630 proteins by blastx, and a CDS feature in the query genome was created when a hit of over 90% identity was found. Glimmer3 [62] was used to predict CDSs in genomic regions where no significant hits were found. Any unique genomic regions left were examined and annotated manually in Artemis [63]. The genome comparisons were visualized in Artemis and ACT (Artemis Comparison Tool) [64]. The sequences of CD196 and R20291 can be accessed using the accession numbers [EMBL: FN538970] and [EMBL: FN545816], respectively.

The reciprocal-best-hit fasta search algorithm was used to identify orthologues among strains 630, CD196 and R20291. All CDSs in the query genome were searched in the database of subject CDSs by FASTA [65]. When a hit of over 30% identity and over 80% length was found, the hit CDS in the subject genome was searched again in the database of query CDSs in a similar fashion. If the hit of the second search is the same as the original query CDS, the two CDSs are considered as orthologues by this method. These identified orthologues were manually curated to account for inaccuracies caused by inserted elements, frameshifts and pseudogenes.

### Toxin B toxicity assay

Toxins were produced by the dialyzing cultivation method [66] with BHI broth (Oxoid) as outer medium and 10% NaCl as inner medium. Cultures were performed at 37°C for 4 days. Toxin B was purified as previously described [67] using ion exchange chromatography (DEAE-Sephacel, GE Healthcare Life Sciences, Little Chalfont, Buckinghamshire UK) and gel filtration (Superdex G200, GE Healthcare Life Sciences). Toxin B preparations were analyzed by SDS-PAGE and the band corresponding to toxin B for each strain was quantified by gel densitometry (Additional data file 4).

Cytotoxicity assays were performed as previously described [68]. Subconfluent cell monolayers were obtained in 96 well plates and were inoculated with serial dilutions of toxin B samples. The cells were monitored for 24 hours after inoculation for morphological alteration. The cytotoxicity titer corresponds to the reciprocal of the greater dilution giving rounding up in 50% of the cells and is expressed as toxin molarity (corresponding to toxin specific activity).

### Motility assay

Cultures were grown anaerobically for 1 to 2 days on Braziers media from glycerol stocks. BHI agar (0.05%) was poured into 30 ml glass vials that were then pre-equilibrated for 4 hours in the anaerobe chamber. Three single colonies were picked with a loop and inoculated into the top 2 to 5 mm of BHI agar in the glass vial. These were then left overnight in the anaerobe chamber; the vials were then removed from the anaerobe chamber and photographed to record the motility.

### Minimum inhibitory concentration determination using Etest

Chloramphenicol, erythromycin, tetracycline and fluoroquinolones (gatifloxacin, mocifloxacin and lexofloxacin) MICs were determined using Etest strips (Biomérieux, Marcy l'Etoile, France). *C. difficile *was cultured overnight on Brazier's CCEY agar (Bioconnections) with 1% defibrinated horse blood (Oxoid) and *C. difficile *supplement (Bioconnections). All cultures were undertaken at 37°C in an anaerobe chamber (10% CO_2_, 10% H_2_, 80% N_2_). A bacterial suspension in BHI (no. 3 McFarland standard) was inoculated onto the surface of Brazier's agar and was then dried for 15 to 30 minutes. Etest strips were placed onto agar surface. Agar plates were incubated anaerobically (37°C) for 24 hours and 48 hours, and MICs were determined following the manufacturer's instructions.

### Statistical analysis

Chloramphenicol, erythromycin, tetracycline and fluoroquinolone MICs for *C. difficile *630, CD196 and R20291 were analyzed by Tukey test using GraphPad Prism 4 software (La Jolla, CA, USA). *P*-value < 0.05 was considered statistically significant.

## Abbreviations

BHI: brain heart infusion; CDI: *C. difficile *infection; CDS: coding sequence; IS: Insertion Sequence; MIC: minimum inhibitory concentration; PaLoc: pathogenicity locus; PMC: pseudomembranous colitis; SMPI: Stoke Mandeville phage island.

## Authors' contributions

RAS, JP, GD and BWW conceived of the study. MAQ and GR performed the sequencing experiments. MH, CC, MS, TDL and JP performed the data analysis. MG and MRP performed toxin B toxicity assays, LD performed motility and autoagglutination assays, EV performed MIC assays, MM performed PCR analysis, and DNG provided strains and critical analysis. RAS, LD, JP, MH and BWW drafted the manuscript. All authors contributed to and approved the final manuscript.

## Additional data files

The following additional data are available with the online version of this paper: CDSs specific to PCR-ribotype 027 isolates (Additional data file 1); CDSs that have been disrupted by an insertion in both 027 strains but are intact in 630, and CDSs that are intact in both 027 strains but have been disrupted in 630 (Additional data file 2); R20291-specific gene primers used in this study (Additional data file 3); SDS-PAGE of toxin B preparations (Additional data file 4).

## Supplementary Material

Additional data file 1 CDS specific to PCR-ribotype 027 isolates. Click here for file

Additional data file 2 Fourteen CDSs have been disrupted by an insertion in both 027 strains but are intact in 630, and, conversely, 12 CDSs are intact in both 027 strains but have been disrupted in 630. Click here for file

Additional data file 3 R20291-specific gene primers used in this study. Click here for file

Additional data file 4 Toxin B was quantified by gel densitometry. 1 = VPI10463, 2 = CD196, 3 = 630. Click here for file
