# Supplementary material for: Comparative genome and phenotypic analysis of Clostridium difficile 027 strains provides insight into the evolution of a hypervirulent bacterium
Source: Genome Biol. 2009 Sep 25;10(9):R102. doi: 10.1186/gb-2009-10-9-r102 (PMC2768977; doi:10.1186/gb-2009-10-9-r102)
Supplement: Additional data file 1 — CDS specific to PCR-ribotype 027 isolates. [file gb-2009-10-9-r102-S1.doc]

Supplementary table 1 CDS specific to PCR-ribotype 027 isolates

| **R20291** | **CD196** | **Fn Cat** | **Function** |
| --- | --- | --- | --- |
| CDR20291_0043 | CD196_0055 | 3.7.2 | thymidylate synthase |
| CDR20291_0044 | CD196_0056 | 3.7.0 | dihydrofolate reductase region |
| CDR20291_0045 | CD196_0057 | 0.0.2 | putative uncharacterized protein |
| CDR20291_0046 | CD196_0058 | 3.2.14 | thiamine biosynthesis protein thic |
| CDR20291_0047 | CD196_0059 | 3.7.2 | putative thymidylate synthase |
| CDR20291_0200 | CD196_0213 | 0.0.2 | putative uncharacterized protein |
| CDR20291_0201 | CD196_0214 | 0.0.2 | putative uncharacterized protein |
| CDR20291_0223 | CD196_0236 | 3.3.18 | DTDP-4-dehydrorhamnose reductase |
| CDR20291_0224 | CD196_0237 | 3.3.18 | glucose-1-phosphate thymidylyltransferase |
| CDR20291_0225 | CD196_0238 | 3.3.18 | DTDP -4-dehydrorhamnose 3,5-epimerase |
| CDR20291_0226 | CD196_0239 | 3.3.18 | DTDP -glucose 4,6-dehydratase |
| CDR20291_0242 | CD196_0255 | 2.2.8 | glycosyl transferase family 2 |
| CDR20291_0243 | CD196_0256 | 2.2.8 | glycosyl transferase, group 2 family |
| CDR20291_0244 | CD196_0257 | 0.0.2 | putative uncharacterized protein |
| CDR20291_0245 | CD196_0258 | 7.0.0 | putative carbamoyl-phosphate-synthetase |
| CDR20291_0246 | CD196_0259 | 3.1.16 | putative ornithine cyclodeaminase |
| CDR20291_0247 | CD196_0260 | 0.0.2 | putative uncharacterized protein |
| CDR20291_0309 | CD196_0322 | 0.0.2 | putative uncharacterized protein |
| CDR20291_0310 | CD196_0323 | 2.2.11 | tetR (putative transcriptional regulator) |
| CDR20291_0377 | CD196_0390 | 5.1.4 | transposase-like protein b |
| CDR20291_0382 | CD196_0396 | 5.1.4 | integrase, catalytic region |
| CDR20291_0383 | CD196_0397 | 0.0.2 | putative uncharacterized protein |
| CDR20291_0405 | CD196_0419 | 5.1.4 | transposase-like protein b |
| CDR20291_0427 | CD196_0441 | 0.0.2 | putative uncharacterized protein |
| CDR20291_0438 | CD196_0452 | 6.1.2 | dna-binding response regulator |
| CDR20291_0439 | CD196_0453 | 6.1.1 | sensor histidine kinase |
| CDR20291_0440 | CD196_0454 | 4.1.6 | cell surface protein (putative hemagglutinin/adhesin) |
| CDR20291_0500 | CD196_0516 | 5.1.4 | putative uncharacterized protein |
| CDR20291_0501 | CD196_0518 | 0.0.2 | putative uncharacterized protein |
| CDR20291_0502 | CD196_0519 | 0.0.2 | putative uncharacterized protein |
| CDR20291_0510 | CD196_0527 | 6.5.0 | putative signaling protein |
| CDR20291_0514 | CD196_0530 | 5.1.4 | transposase-like protein b |
| CDR20291_0528 | CD196_0544 | 0.0.2 | putative uncharacterized protein |
| CDR20291_0530 | CD196_0546 | 5.1.4 | transposase-like protein b |
| CDR20291_0531 | CD196_0547 | 2.1.4 | putative membrane-associated metalloprotease |
| CDR20291_0551 | CD196_0569 | 1.5.0 | abc transporter, atp-binding/permease protein |
| CDR20291_0569 | CD196_0587 | 7.0.0 | fic family protein; putative filamentation induced by cAMP protein |
| CDR20291_0601 | CD196_0619 | 4.1.6 | putative membrane protein |
| CDR20291_0602 | CD196_0620 | 4.1.6 | putative membrane protein |
| CDR20291_0603 | CD196_0621 | 3.4.2 | amidohydrolase 3 |
| CDR20291_0604 | CD196_0622 | 0.0.2 | putative uncharacterized protein |
| CDR20291_0667 | CD196_0686 | 0.0.0 | [no /product] |
| CDR20291_0697 | CD196_0716 | 5.1.4 | transposase-like protein b |
| CDR20291_0710 | CD196_0729 | 0.0.0 | [no /product] |
| CDR20291_0711 | CD196_0730 | 3.3.11 | cytidine/deoxycytidylate deaminase family protein |
| CDR20291_0809 | CD196_0829 | 5.1.4 | transposase-like protein b |
| CDR20291_0826 | CD196_0845 | 5.1.4 | 3-isopropylmalate dehydrogenase |
| CDR20291_0860 | CD196_0880 | 0.0.2 | putative uncharacterized protein |
| CDR20291_0861 | CD196_0881 | 6.1.0 | sensor protein |
| CDR20291_0862 | CD196_0882 | 1.5.0 | predicted abc transporter, atpase component |
| CDR20291_0863 | CD196_0883 | 0.0.2 | putative uncharacterized protein |
| CDR20291_0959 | CD196_0981 | 5.1.4 | transposase-like protein b |
| CDR20291_0961 | CD196_0983 | 0.0.2 | putative uncharacterized protein |
| CDR20291_0962 | CD196_0984 | 0.0.2 | putative uncharacterized protein |
| CDR20291_0963 | CD196_0985 | 0.0.2 | putative uncharacterized protein |
| CDR20291_0979 | CD196_1001 | 0.0.0 | [no /product] |
| CDR20291_1071 | CD196_1093 | 0.0.2 | putative uncharacterized protein |
| CDR20291_1072 | CD196_1094 | 5.1.4 | integrase, catalytic region |
| CDR20291_1078 | CD196_1100 | 0.0.0 | [no /product] |
| CDR20291_1184 | CD196_1206 | 0.0.0 | [no /product] |
| CDR20291_1277 | CD196_1300 | 0.0.2 | putative uncharacterized protein |
| CDR20291_1278 | CD196_1301 | 6.3.2 | transcriptional regulator, araC family protein |
| CDR20291_1324 | CD196_1347 | 0.0.2 | putative uncharacterized protein |
| CDR20291_1338 | CD196_1361 | 5.1.4 | transposase-like protein b |
| CDR20291_1415 | CD196_1438 | 5.1.2 | prophage lambdaba04, site-specific recombinase, phage integrase family |
| CDR20291_1416 | CD196_1439 | 0.0.2 | putative uncharacterized protein |
| CDR20291_1417 | CD196_1440 | 1.7.1 | hipa-like |
| CDR20291_1418 | CD196_1441 | 5.1.2 | putative phage-related protein |
| CDR20291_1420 | CD196_1445 | 0.0.2 | putative uncharacterized protein; putative uncharacterized protein |
| CDR20291_1421 | CD196_1446 | 0.0.2 | putative uncharacterized protein |
| CDR20291_1422 | CD196_1447 | 0.0.0 | [no /product] |
| CDR20291_1423 | CD196_1448 | 0.0.2 | putative uncharacterized protein |
| CDR20291_1424 | CD196_1449 | 2.2.3 | DNA-directed DNA polymerase I |
| CDR20291_1425 | CD196_1450 | 7.0.0 | putative virulence-associated protein e |
| CDR20291_1426 | CD196_1451 | 6.5.0 | snf2-related protein |
| CDR20291_1427 | CD196_1452 | 0.0.2 | putative uncharacterized protein |
| CDR20291_1428 | CD196_1453 | 6.5.0 | bro family, n-terminal |
| CDR20291_1429 | CD196_1454 | 0.0.0 | [no /product] |
| CDR20291_1430 | CD196_1455 | 0.0.0 | [no /product] |
| CDR20291_1431 | CD196_1456 | 5.1.2 | putative phage DNA-binding protein |
| CDR20291_1432 | CD196_1457 | 5.1.2 | phage terminase large subunit |
| CDR20291_1433 | CD196_1458 | 5.1.2 | phage portal protein |
| CDR20291_1434 | CD196_1459 | 5.1.2 | phage protein |
| CDR20291_1435 | CD196_1460 | 5.1.2 | scaffold protein |
| CDR20291_1436 | CD196_1461 | 5.1.2 | putative phage major capsid protein |
| CDR20291_1437 | CD196_1462 | 5.1.2 | hypothetical phage protein |
| CDR20291_1438 | CD196_1463 | 5.1.2 | phage protein |
| CDR20291_1439 | CD196_1464 | 0.0.2 | putative uncharacterized protein |
| CDR20291_1440 | CD196_1465 | 5.1.2 | phage protein |
| CDR20291_1441 | CD196_1466 | 5.1.2 | phage protein |
| CDR20291_1442 | CD196_1467 | 5.1.2 | phage protein |
| CDR20291_1443 | CD196_1468 | 5.1.2 | phage-related protein |
| CDR20291_1444 | CD196_1469 | 5.1.2 | phage protein |
| CDR20291_1445 | CD196_1470 | 5.1.2 | hypothetical phage protein |
| CDR20291_1446 | CD196_1471 | 5.1.2 | prophage antirepressor-related protein |
| CDR20291_1447 | CD196_1472 | 0.0.2 | putative uncharacterized protein |
| CDR20291_1448 | CD196_1473 | 0.0.2 | putative uncharacterized protein |
| CDR20291_1449 | CD196_1474 | 5.1.2 | putative phage tail tape measure protein |
| CDR20291_1450 | CD196_1475 | 5.1.2 | putative phage cell wall hydrolase |
| CDR20291_1451 | CD196_1476 | 5.1.2 | putative phage cell wall hydrolase |
| CDR20291_1452 | CD196_1477 | 5.1.2 | phage protein |
| CDR20291_1453 | CD196_1478 | 5.1.2 | phage protein |
| CDR20291_1454 | CD196_1479 | 5.1.2 | putative phage tail protein |
| CDR20291_1455 | CD196_1480 | 5.1.2 | phage protein |
| CDR20291_1456 | CD196_1481 | 5.1.2 | putative phage tail fiber protein |
| CDR20291_1457 | CD196_1482 | 5.1.2 | hypotheticalphage protein |
| CDR20291_1458 | CD196_1483 | 5.1.2 | putative uncharacterized protein |
| CDR20291_1459 | CD196_1484 | 5.1.2 | hypothetical phage protein |
| CDR20291_1460 | CD196_1485 | 0.0.2 | putative uncharacterized protein |
| CDR20291_1461 | CD196_1486 | 5.1.2 | putative uncharacterized protein |
| CDR20291_1462 | CD196_1487 | 0.0.2 | putative uncharacterized protein |
| CDR20291_1463 | CD196_1488 | 5.1.2 | n-acetylmuramoyl-l-alanine amidase (cell wall hydrolase) |
| CDR20291_1464 | CD196_1489 | 1.4.3 | cell surface protein (putative penicillin-binding protein) |
| CDR20291_1469 | CD196_1494 | 5.1.4 | transposase-like protein b |
| CDR20291_1847 | CD196_1802 | 7.0.0 | possible transcriptional regulator |
| CDR20291_1848 | CD196_1803 | 2.1.4 | putative peptidase |
| CDR20291_1849 | CD196_1805 | 1.5.1 | putative aminobenzoyl-glutamate transport protein |
| CDR20291_1850 | CD196_1806 | 7.0.0 | putative aminotransferase |
| CDR20291_1906 | CD196_1863 | 5.1.4 | transposase-like protein b |
| CDR20291_1923 | CD196_1880 | 6.3.12 | hth transcriptional regulator merR family |
| CDR20291_1924 | CD196_1881 | 0.0.2 | putative uncharacterized protein |
| CDR20291_1948 | CD196_1905 | 0.0.2 | putative uncharacterized protein |
| CDR20291_1949 | CD196_1906 | 0.0.2 | putative uncharacterized protein |
| CDR20291_2092 | CD196_2049 | 4.1.7 | putative lipoprotein |
| CDR20291_2183 | CD196_2140 | 1.4.3 | putative beta-lactamase inducer |
| CDR20291_2196 | CD196_2150 | 5.1.4 | integrase, catalytic region |
| CDR20291_2197 | CD196_2151 | 5.1.4 | transposase |
| CDR20291_2198 | CD196_2152 | 0.0.0 | hypothetical protein |
| CDR20291_2275 | CD196_2228A | 4.1.9 | putative beta-lactamase inducer |
| CDR20291_2278 | CD196_2230 | 4.1.9 | putative peptidoglycan- binding/hydrolysing protein |
| CDR20291_2279 | CD196_2231 | 1.4.0 | putative beta-lactamase inducer |
| CDR20291_2280 | CD196_2232 | 1.4.0 | putative beta-lactamase repressor |
| CDR20291_2299 | CD196_2252 | 5.1.4 | transposase |
| CDR20291_2491 | CD196_2444 | 5.1.5 | cdta (adp-ribosyltransferase enzymatic component) |
| CDR20291_2492 | CD196_2445 | 5.1.5 | adp-ribosyltransferase binding component |
| CDR20291_2501 | CD196_2454 | 5.1.4 | transposase-like protein b |
| CDR20291_2514 | CD196_2467 | 0.0.2 | putative uncharacterized protein |
| CDR20291_2515 | CD196_2468 | 1.5.1 | amino acid permease family protein |
| CDR20291_2516 | CD196_2469 | 2.1.4 | cobalt dependent x-pro dipeptidase |
| CDR20291_2517 | CD196_2470 | 6.5.0 | putative transcriptional regulator |
| CDR20291_2674 | CD196_2627 | 4.1.6 | putative membrane protein |
| CDR20291_2709 | CD196_2662 | 5.1.4 | transposase |
| CDR20291_2716 | CD196_2669 | 5.1.4 | transposase-like protein b |
| CDR20291_2757 | CD196_2710 | 0.0.2 | putative uncharacterized protein |
| CDR20291_2758 | CD196_2711 | 1.4.1 | putative lantibiotic ABC transporter, ATP-binding protein |
| CDR20291_2759 | CD196_2712 | 0.0.0 | sortase |
| CDR20291_2760 | CD196_2713 | 6.1.1 | two-component system, sensor histidine kinas |
| CDR20291_2761 | CD196_2714 | 0.0.2 | putative uncharacterized protein |
| CDR20291_2834 | CD196_2786 | 5.1.4 | transposase-like protein b |
| CDR20291_2908 | CD196_2861 | 0.0.2 | putative uncharacterized protein |
| CDR20291_2909 | CD196_2862 | 2.1.3 | type I restriction enzyme r subunit |
| CDR20291_2910 | CD196_2863 | 7.0.0 | hypothetical transmembrane protein |
| CDR20291_2911 | CD196_2864 | 2.1.3 | restriction modification system dna specificity domain |
| CDR20291_2912 | CD196_2865 | 2.2.3 | type I restriction enzyme m subunit |
| CDR20291_2950 | CD196_2902 | 5.1.4 | transposase-like protein b |
| CDR20291_2959 | CD196_2912 | 5.1.4 | conjugative transposon site-specific recombinase |
| CDR20291_2960 | CD196_2913 | 0.0.2 | putative uncharacterized protein |
| CDR20291_2961 | CD196_2914 | 0.0.2 | putative uncharacterized protein |
| CDR20291_2962 | CD196_2915 | 3.5.0 | predicted enoate reductase |
| CDR20291_2963 | CD196_2916 | 1.5.0 | nitrate/nitrite transporter |
| CDR20291_2964 | CD196_2917 | 6.3.14 | transcriptional regulator, padR-like family |
| CDR20291_2980 | CD196_2933 | 1.5.0 | probable abc transporter permease protein y4fn |
| CDR20291_2981 | CD196_2934 | 1.5.0 | spermidine/putrescine abc transporter atp-binding subunit |
| CDR20291_2982 | CD196_2935 | 0.0.2 | putative uncharacterized protein |
| CDR20291_2983 | CD196_2936 | 1.5.0 | ABC-type fe3+ transport system periplasmic component-like protein; |
| CDR20291_2986 | CD196_2939 | 0.0.2 | putative uncharacterized protein |
| CDR20291_2987 | CD196_2940 | 0.0.2 | putative uncharacterized protein |
| CDR20291_2988 | CD196_2941 | 0.0.2 | putative uncharacterized protein |
| CDR20291_2989 | CD196_2942 | 0.0.2 | putative uncharacterized protein |
| CDR20291_2990 | CD196_2943 | 0.0.2 | putative uncharacterized protein |
| CDR20291_2991 | CD196_2944 | 6.5.0 | frg domain protein |
| CDR20291_2992 | CD196_2945 | 5.1.4 | transposase, is4 |
| CDR20291_2993 | CD196_2946 | 0.0.2 | putative uncharacterized protein |
| CDR20291_2994 | CD196_2947 | 1.4.0 | crispr-associated helicase cas3 |
| CDR20291_2995 | CD196_2948 | 1.4.0 | crispr-associated autoregulator, devR family |
| CDR20291_2996 | CD196_2949 | 0.0.2 | putative uncharacterized protein |
| CDR20291_2997 | CD196_2950 | 1.4.0 | crispr-associated protein cas6 |
| CDR20291_2998 | CD196_2951 | 1.4.0 | crispr-associated protein cas5 family |
| CDR20291_2999 | CD196_2952 | 0.0.2 | putative uncharacterized protein |
| CDR20291_3000 | CD196_2953 | 5.1.2 | putative phage-related replicative helicase |
| CDR20291_3010 | CD196_2963 | 5.1.2 | phage-related protein |
| CDR20291_3025 | CD196_2978 | 2.2.10 | gcn5-related n-acetyltransferase; gcn5-related n-acetyltransferase |
| CDR20291_3049 | CD196_3003 | 1.5.0 | abc transporter, atp-binding protein |
| CDR20291_3050 | CD196_3004 | 1.5.0 | abc transporter, permease associated with salivaricin lantibiotic |
| CDR20291_3051 | CD196_3005 | 6.1.1 | possible sensor histidine kinase |
| CDR20291_3052 | CD196_3006 | 6.1.2 | salavaricin two-component response regulator |
| CDR20291_3123 | CD196_3077 | 1.5.0 | ABC transporter, ATP-binding protein |
| CDR20291_3124 | CD196_3078 | 6.1.1 | sensor protein |
| CDR20291_3125 | CD196_3079 | 0.0.2 | putative uncharacterized protein |
| CDR20291_3184 | CD196_3138 | 4.1.6 | putative membrane protein |
| CDR20291_3185 | CD196_3139 | 1.5.0 | abc transporter, atp-binding protein |
| CDR20291_3186 | CD196_3140 | 4.1.6 | membrane protein, putative |
| CDR20291_3187 | CD196_3141 | 6.5.0 | accessory gene regulator |
| CDR20291_3188 | CD196_3142 | 6.1.1 | sensor histidine kinase virS |
| CDR20291_3189 | CD196_3143 | 6.1.2 | DNA-binding response regulator, lyttr family |
| CDR20291_3276 | CD196_3230 | 5.1.4 | transposase, mutator type |
| CDR20291_3277 | CD196_3231 | 4.1.7 | putative exported protein |
| CDR20291_3278 | CD196_3232 | 4.1.7 | putative exported protein; |
| CDR20291_3279 | CD196_3233 | 6.5.0 | putative regulatory protein |
| CDR20291_3280 | CD196_3234 | 0.0.2 | putative uncharacterized protein |
| CDR20291_3281 | CD196_3235 | 5.1.4 | transposon tn21 resolvase |
| CDR20291_3282 | CD196_3236 | 0.0.2 | putative uncharacterized protein |
| CDR20291_3283 | CD196_3237 | 0.0.2 | putative uncharacterized protein |
| CDR20291_3284 | CD196_3238 | 5.1.2 | phage portal protein; phage portal protein |
| CDR20291_3285 | CD196_3239 | 0.0.2 | putative uncharacterized protein |
| CDR20291_3286 | CD196_3240 | 0.0.2 | putative uncharacterized protein |
| CDR20291_3287 | CD196_3241 | 0.0.2 | putative uncharacterized protein |
| CDR20291_3288 | CD196_3242 | 0.0.2 | putative uncharacterized protein |
| CDR20291_3289 | CD196_3243 | 7.0.0 | leucine-rich repeat protein |
| CDR20291_3290 | CD196_3244 | 5.1.4 | transposase mutator type |
| CDR20291_3453 | CD196_3407 | 5.1.5 | putative collagen-binding surface protein |
| CDR20291_3454 | CD196_3408 | 5.1.4 | conjugative transposon protein |
| CDR20291_3455 | CD196_3409 | 0.0.2 | putative uncharacterized protein |
| CDR20291_3456 | CD196_3410 | 0.0.2 | putative uncharacterized protein |
| CDR20291_3457 | CD196_3411 | 0.0.2 | putative uncharacterized protein |
| CDR20291_3458 | CD196_3412 | 5.1.4 | putative conjugative transposon FtsK_SpoIIIE-related protein |
| CDR20291_3459 | CD196_3413 | 5.1.4 | putative conjugative transposon replication initiation factor |
| CDR20291_3460 | CD196_3414 | 5.1.4 | conjugative transposon protein |
| CDR20291_3461 | CD196_3415 | 1.4.3 | chloramphenicol o-acetyltransferase |
| CDR20291_3462 | CD196_3416 | 5.1.4 | conjugative transposon protein |
| CDR20291_3463 | CD196_3417 | 5.1.4 | conjugative transposon protein |
| CDR20291_3464 | CD196_3418 | 5.1.4 | conjugative transposon protein |
| CDR20291_3465 | CD196_3419 | 5.1.4 | conjugative transposon protein |
| CDR20291_3466 | CD196_3420 | 5.1.4 | conjugative transposon protein |
| CDR20291_3467 | CD196_3421 | 5.1.4 | conjugative transposon protein |
| CDR20291_3468 | CD196_3422 | 0.0.2 | putative uncharacterized protein |
| CDR20291_3469 | CD196_3423 | 6.5.0 | transcriptional regulator, tetR family |
| CDR20291_3470 | CD196_3424 | 1.5.0 | abc transporter, atp-binding protein |
| CDR20291_3471 | CD196_3425 | 1.5.0 | putative abc transporter, permease protein |
| CDR20291_3472 | CD196_3426 | 1.5.0 | putative abc transporter, permease protein |
| CDR20291_3473 | CD196_3427 | 0.0.2 | putative uncharacterized protein |
| CDR20291_3474 | CD196_3428 | 0.0.2 | putative uncharacterized protein |
| CDR20291_3475 | CD196_3429 | 5.1.4 | conjugative transposon protein |
| CDR20291_3478 | CD196_3432 | 5.1.4 | transposase-like protein b |
