# Supplementary material for: Comparative genome and phenotypic analysis of Clostridium difficile 027 strains provides insight into the evolution of a hypervirulent bacterium
Source: Genome Biol. 2009 Sep 25;10(9):R102. doi: 10.1186/gb-2009-10-9-r102 (PMC2768977; doi:10.1186/gb-2009-10-9-r102)
Supplement: Additional data file 3 — R20291-specific gene primers used in this study. [file gb-2009-10-9-r102-S3.docx]

Additional data file 4. R20291 Specific gene primers used in this study
