# Supplementary figures and images for: Comparative genome and phenotypic analysis of Clostridium difficile 027 strains provides insight into the evolution of a hypervirulent bacterium
Source: Genome Biol. 2009 Sep 25;10(9):R102. doi: 10.1186/gb-2009-10-9-r102 (PMC2768977; doi:10.1186/gb-2009-10-9-r102)

## Slide 1
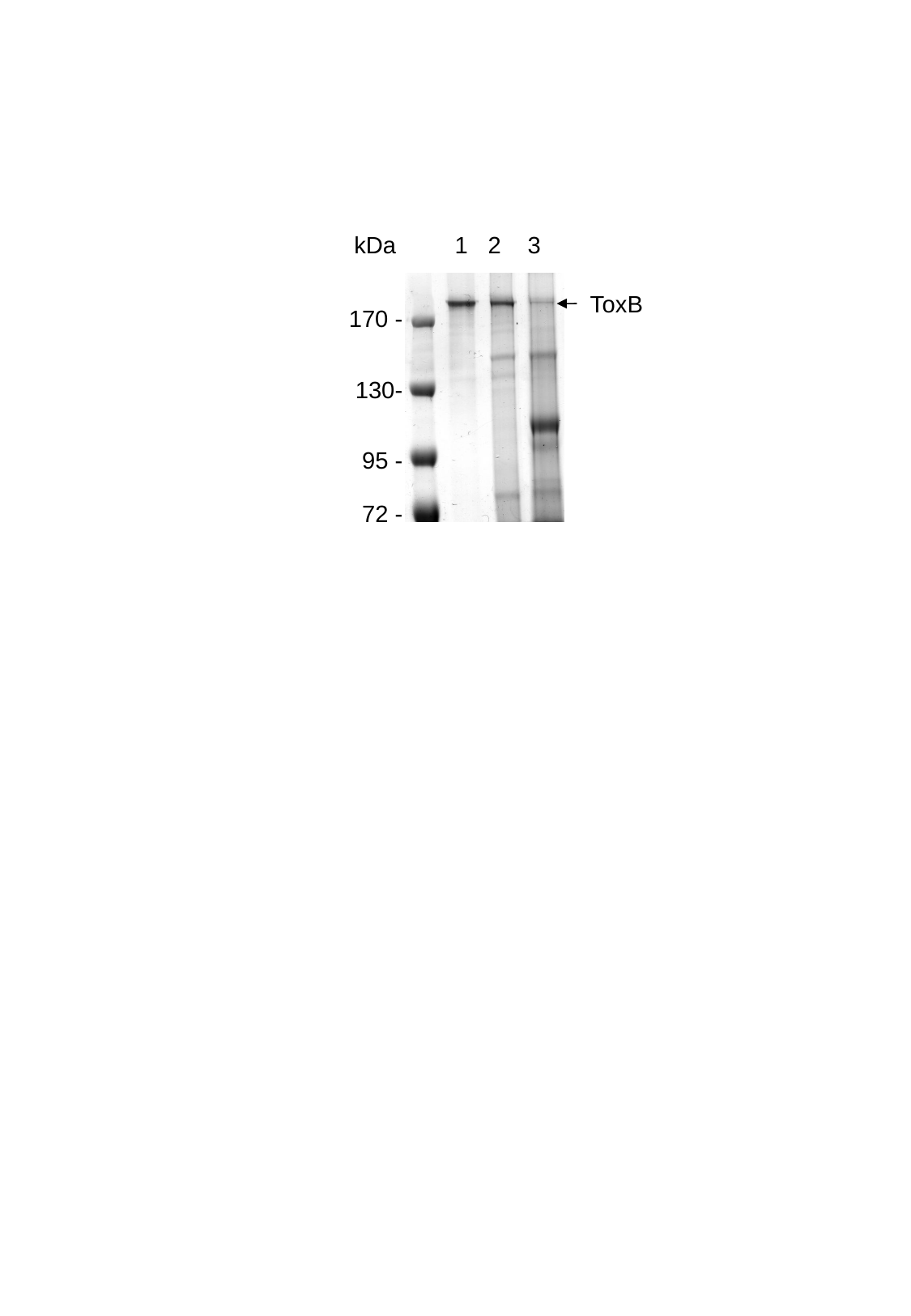

kDa
1 2 3
ToxB
170 -
130-
95 -
72 -

Supplement: Additional data file 4 — Toxin B was quantified by gel densitometry. 1 = VPI10463, 2 = CD196, 3 = 630. [file gb-2009-10-9-r102-S4.ppt]
